# Supplementary material for: RNA m6A methylation modulates airway inflammation in allergic asthma via PTX3-dependent macrophage homeostasis
Source: Nat Commun. 2023 Nov 13;14:7328. doi: 10.1038/s41467-023-43219-w (PMC10643624; doi:10.1038/s41467-023-43219-w)
Supplement: Supplementary file 1 — Supplementary Information [file 41467_2023_43219_MOESM1_ESM.pdf]

SUPPLEMENTARY INFORMATION

RNA m^{6}A Methylation Modulates Airway Inflammation in Allergic Asthma via PTX3-dependent Macrophage Homeostasis

Authors: Xiao Han[1,2#*], Lijuan Liu[3#], Saihua Huang[1,2#], Wenfeng Xiao[1,2#], Yajing Gao[1,2#], WeiTao Zhou[3], Caiyan Zhang[4], Hongmei Zheng[3], Lan Yang[1,2], Xueru Xie[1,2], Qiuyan Liang[1,2], Zikun Tu[1,2], Hongmiao Yu[1,2], Jinrong Fu[5], Libo Wang[3], Xiaobo Zhang[3], Liling Qian[3,6*], Yufeng Zhou[1,2*]

# These authors contributed equally to this work.

* Corresponding author: Yufeng Zhou, M.D., Ph.D., 399 Wanyuan Rd, Minhang, Shanghai 201102, China, e-mail: yfzhou1@fudan.edu.cn. TEL: 86-21-64932907; Or Liling Qian, M.D., Ph.D., e-mail: llqian@126.com. TEL: 86-21-52971002; Or Xiao Han, Ph.D., e-mail: sqhx12@126.com. TEL: 86-21-64932897.

.

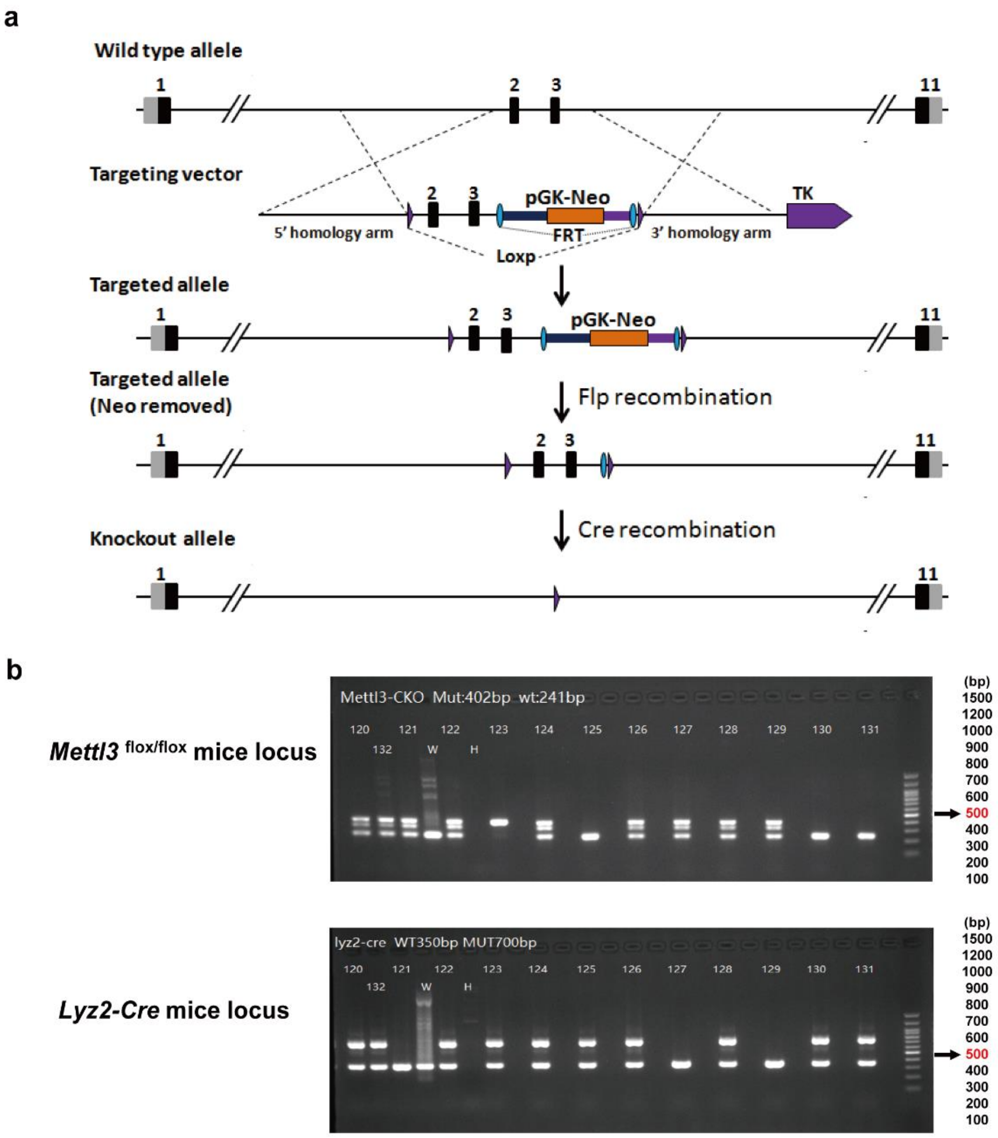

Supplementary Figure 1 Generation of *Mettl3* KO mice. (a) Schematic diagram of the targeting strategy for *Mettl3* depletion in the myeloid compartment, using a LoxP targeting system and homologous recombination. (b) Gel image of PCR products amplified using genotyping primers to identify *Mettl3*$^{fl/fl}$Lyz2$^{Cre/+}$ (KO) mice. W, wide-type (WT); H, ddH_2O; 122, *Mettl3*$^{fl/-}$*Lyz2*$^{Cre/+}$heterozygote (HE); 123, *Mettl3*$^{fl/fl}$*Lyz2*$^{Cre/+}$ (KO).

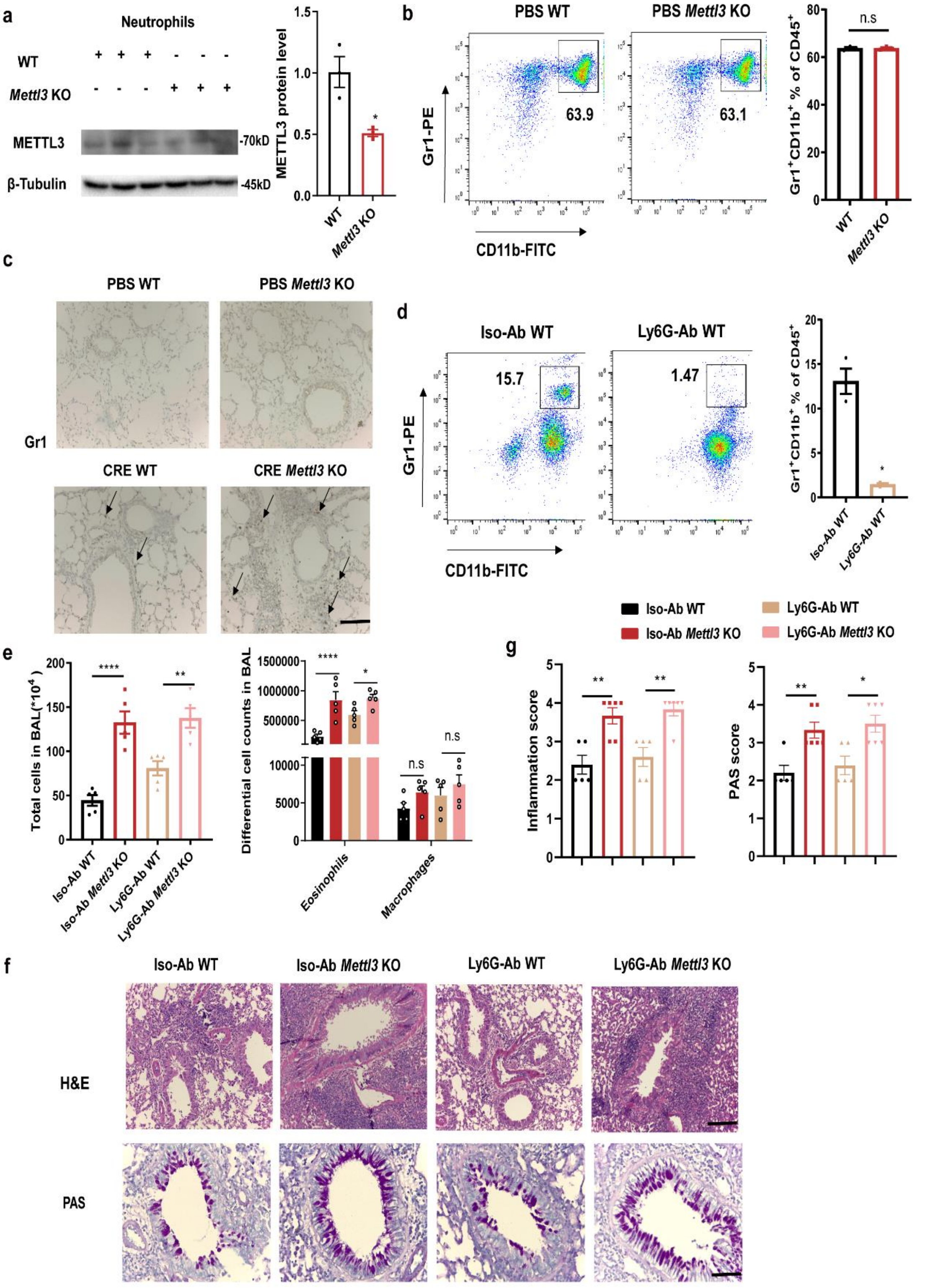

Supplementary Figure 2 Depletion of neutrophils does not reduce the differences in airway inflammation between *Mettl3* KO and WT mice. (a) Western blot showing reduced METTL3 protein levels in neutrophils purified from the bone marrow of the experimental mice (n=3 animals). (b) The percentage of neutrophils from the bone

marrow was detected in *Mettl3* KO and WT mice (n=3 animals). (c) Representative images of Gr1 expression in lung tissues using IHC. Scale bars: 200 μm. Every 72 h during CRE treatment, mice were i.p. injected with 200 μg anti-Ly6G mAb or isotype control mAb, (d) Flow cytometry analysis of the efficiency of neutrophils depletion in BALF (n=3 animals). (e) Total and differential BALF cell numbers, and (f) histopathological changes in the lung tissues were examined (n=5 animals). Scale bars: 200 μm and 100 μm, respectively. (g) Calculated inflammation and PAS scores (n=5 animals for WT groups and n=6 animals for KO groups). Statistical analysis of the data was performed using two-sided unpaired t test with Welch's correction (d) or not (a, b), 1-way ANOVA (e left, g), and 2-way ANOVA (e right) followed by either Tukey's or Sidak's multiple comparison tests. Data are presented as means ± SEM from one of three independent experiments. $*P < 0.05$, $**P < 0.01$, $****P < 0.0001$; n.s = not significant.

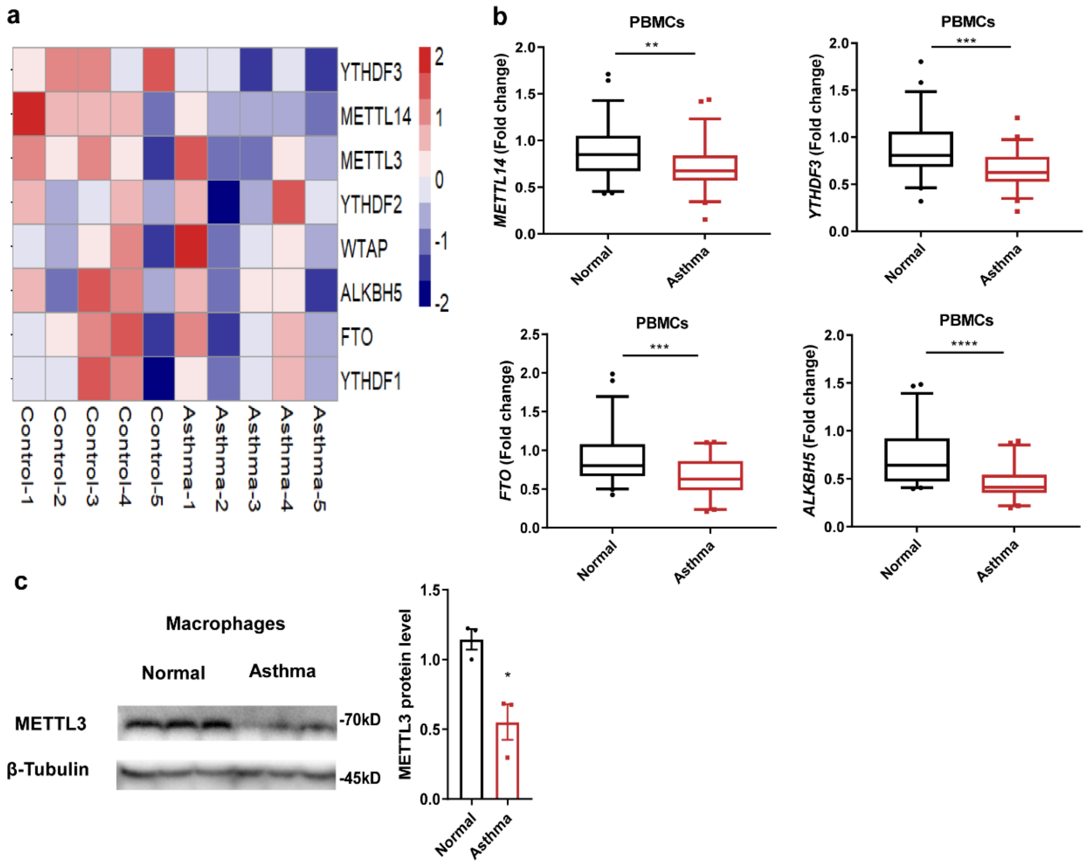

Supplementary Figure 3 Expression of m^{6}A modification-associated genes in childhood allergic asthma patients. (a) Heatmap profiling the expression of m^{6}A modulators in PBMCs of human asthma patients from the GEO database (GSE27876, https://www.ncbi.nlm.nih.gov/geo/query/acc.cgi?acc=GSE27876). (b) RT-qPCR analysis of m^{6}A ‘writer’ (*METTL14*), ‘eraser’ (*FTO* and *ALKBH5*), and ‘reader’ (*YTHDF3*) expression in PBMCs from 55 childhood allergic asthma patients and 50 healthy controls. The detailed minimum, median, maximum, 25th, 75th percentile (box), and 5th and 95th percentile (whiskers) of box plots were provided in the Source data file. (c) The METTL3 protein levels were determined in monocyte-derived macrophages from children with allergic asthma and healthy controls by Western blot (n=3 patients). Statistical analysis of the data was performed using Mann-Whitney test (b left), or two-sided unpaired t test with Welch’s correction (b right) or not (c). Data are presented as means ± SEM. *$P < 0.05$, **$P < 0.01$, ***$P < 0.001$, ****$P < 0.0001$.

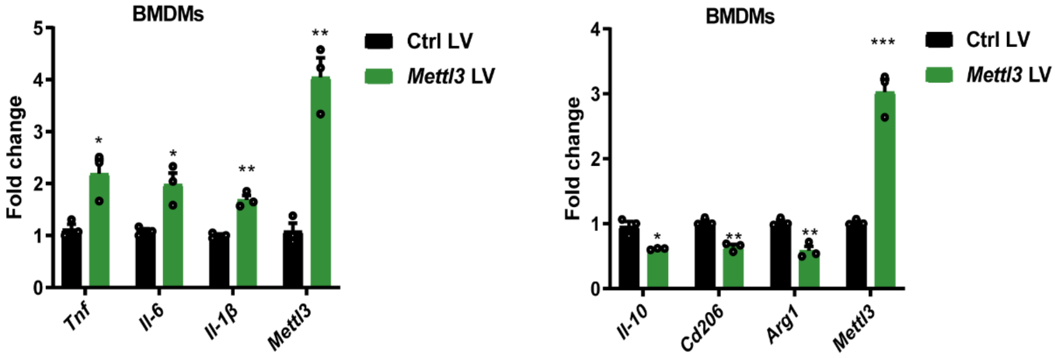

Supplementary Figure 4 Overexpressed METTL3 enhances M1 and inhibits M2 macrophage activation in BMDMs. Overexpression of *Mettl3* in BMDMs with *Mettl3* LV or Ctrl LV. M1 (right)-and M2 (left)-associated markers were quantified by RT-qPCR in macrophages stimulated with LPS or IL-4 (n=3 cells), respectively. Statistical analysis of the data was performed using two-sided unpaired t test. Data are presented as means ± SEM from one of three independent experiments. *$P < 0.05$, **$P < 0.01$, ***$P < 0.001$.

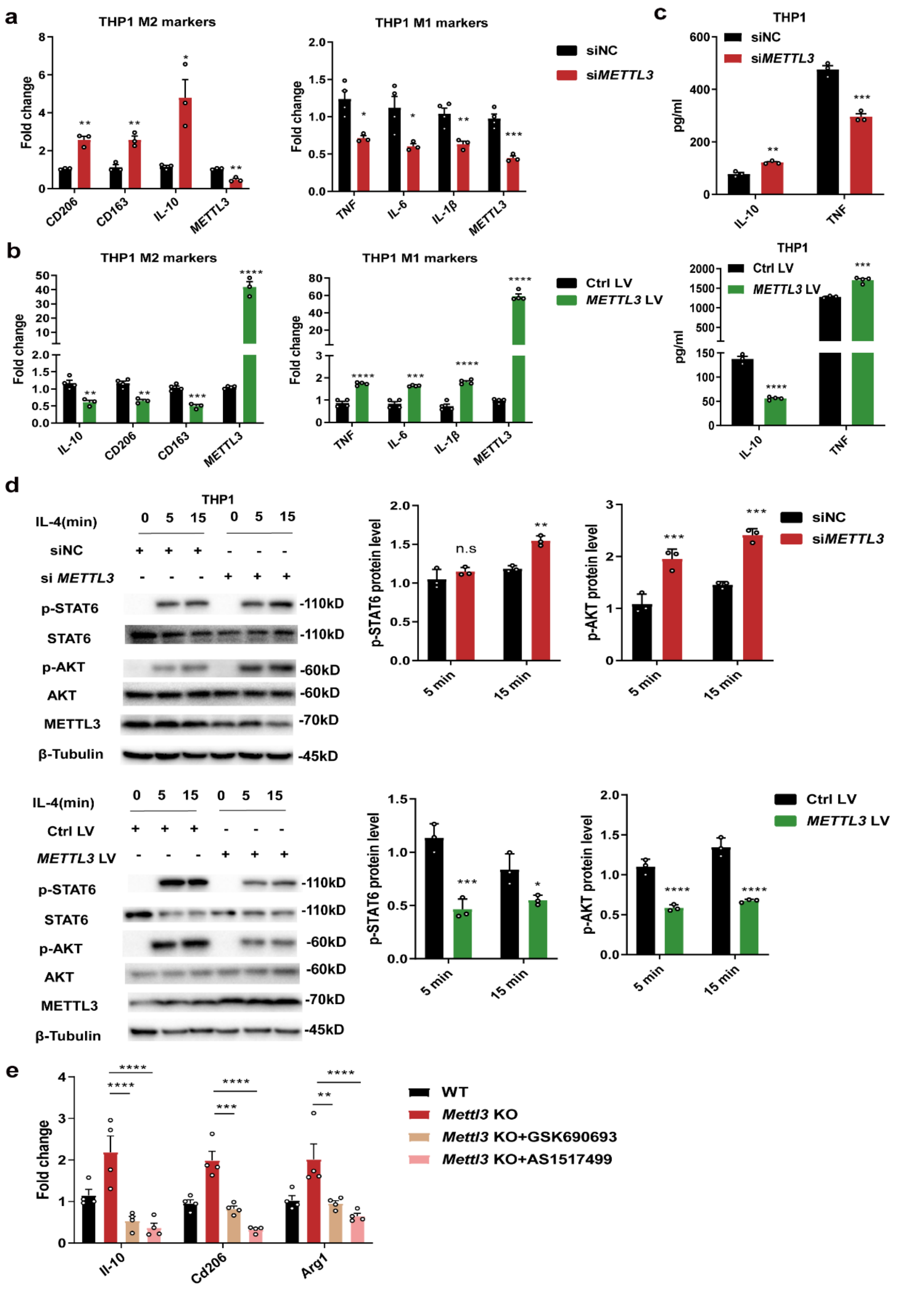

Supplementary Figure 5 METTL3 inhibits M2 macrophage activation via the PI3K/AKT and JAK/STAT6 signaling pathways. (a) Knockdown of *METTL3* in human THP1-derived macrophages using siRNAs pools (200 nM) (n=3 cells), (b) Overexpression of *METTL3* in THP1-derived macrophages with *METTL3* LV or Ctrl

LV (n=4 cells). M1 and M2-associated markers were quantified by RT-qPCR in macrophages stimulated with LPS or IL-4, respectively. NC, negative control. (c) ELISA showing the levels of TNF and IL-10 secretion in THP1-derived macrophages with *METTL3* knockdown (top) (n=3 cells) or overexpression (bottom) (n=3 cells for control group and n=4 cells for overexpression group). (d) Western blot analysis of the levels of AKT phosphorylation (p-AKT), AKT, STAT6, and p-STAT6 in THP1-derived macrophages with *METTL3* knockdown (top) or overexpression (bottom), following IL-4 stimulation (n=3 cells). (e) RT-qPCR detected M2-associated markers expression in BMDMs from WT and *Mettl3* KO mice treated with the AKT inhibitor GSK690693 (100 nM), or the STAT6 inhibitor AS1517499 (100 nM) (n=4 cells). Statistical analysis of the data was performed using two-sided unpaired t test (a-c), and 2-way ANOVA (d-e) followed by either Tukey's or Sidak's multiple comparison tests. Data are presented as means ± SEM from one of three independent experiments. *$P < 0.05$, **$P < 0.01$, ***$P < 0.001$, ****$P < 0.0001$; n.s = not significant.

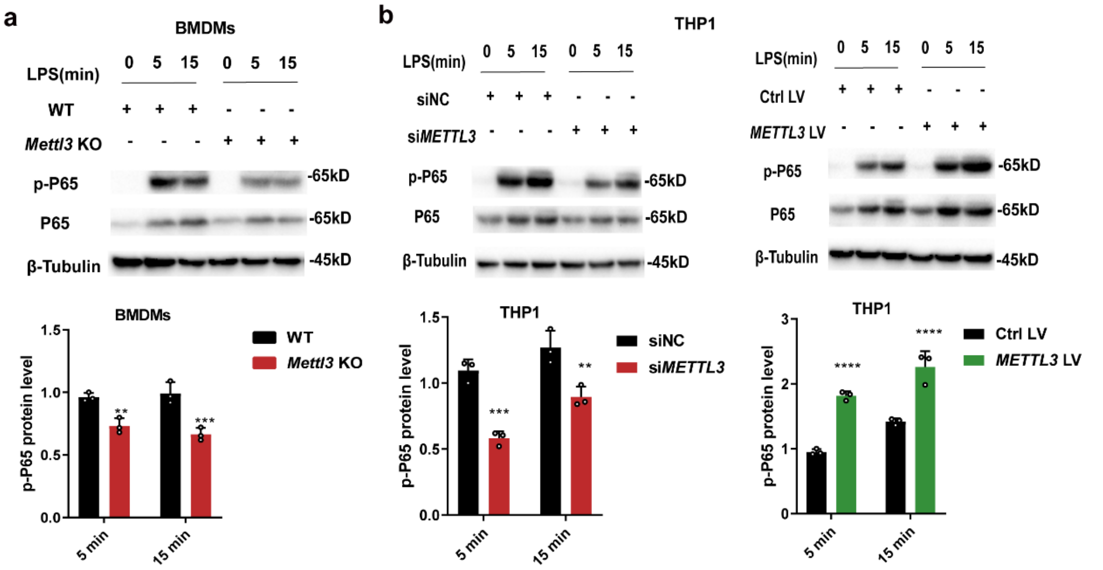

Supplementary Figure 6 METTL3 promotes M1 macrophage activation through the NF-κB signaling pathway. (a) BMDMs from WT and *Mettl3* KO mice (n=3 animals), (b) human THP1-derived macrophages transfected with *METTL3* or control siRNAs (200 nM, left), and *METTL3*-overexpressing (*METTL3* LV, right) THP1-derived macrophages (n=3 cells), stimulated by LPS. Levels of P65 phosphorylation (p-P65) and total P65 were examined by Western blot. Statistical analysis of the data was performed using 2-way ANOVA followed by Sidak's multiple comparison tests. Data are presented as means ± SEM from one of three independent experiments. $^{**}P < 0.01$, $^{***}P < 0.001$, $^{****}P < 0.0001$.

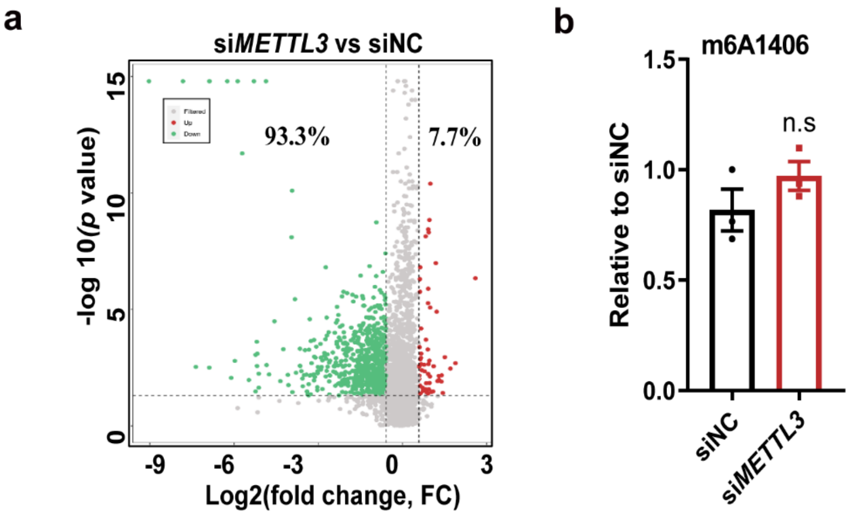

Supplementary Figure 7 Identification m^{6}A levels in METTL3-deficient macrophages. (a) m^{6}A-seq showed significantly increased (red) or decreased (green) m^{6}A peaks (≥ 1.5-fold change, P < 0.05) in human THP1-derived macrophages transfected with *METTL3* siRNA pools, compared to control (NC) cells. (b) Relative amount of SELECT qPCR products targeting the AGA^{1406}CA site on *PTX3* 3'UTR using the total RNA of THP1-derived macrophages transfected with *METTL3* or control siRNA (n=3 cells). Statistical analysis of the data was performed using two-sided unpaired t test (b). Data are presented as means ± SEM from one of three independent experiments. n.s = not significant.

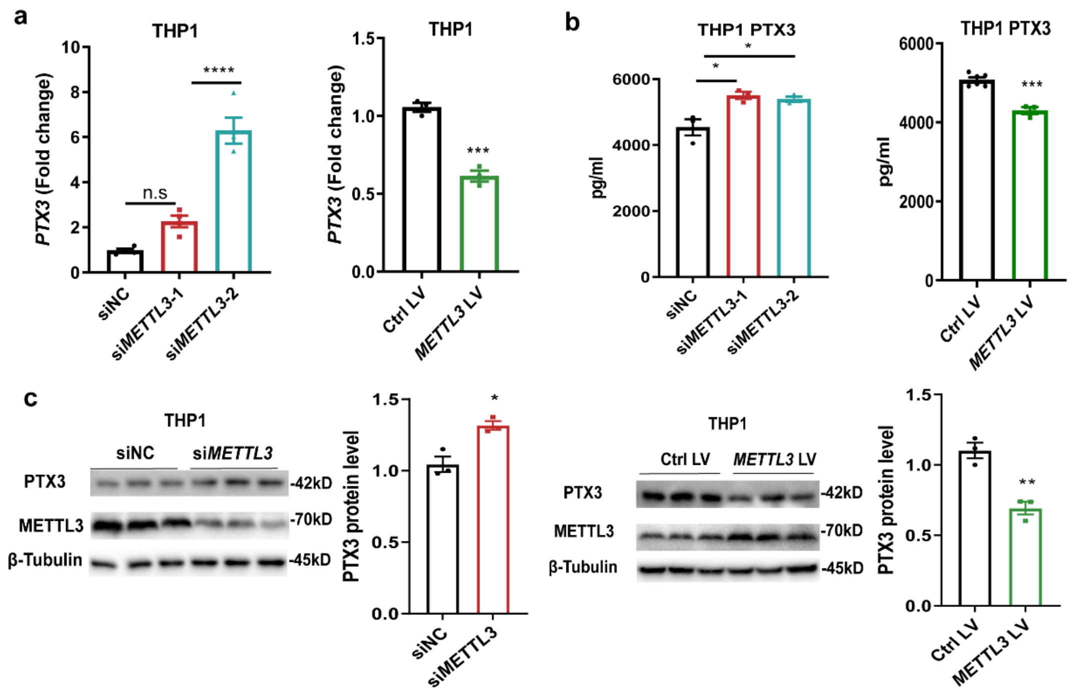

Supplementary Figure 8 METTL3 suppresses *PTX3* expression in human THP1-derived macrophages. After knockdown of *METTL3* in human THP1-derived macrophages using two distinct siRNAs (200 nM, left) or overexpression of *METTL3* in THP1-derived macrophages with *METTL3* LV (right), followed by IL-4 stimulation. mRNA and protein levels of PTX3 were quantified by RT-qPCR (a) (n=4 cells for knockdown group and n=3 cells for overexpression groups), ELISA (b) (n=6 cells for Ctrl LV group and n=3 cells for other groups), and Western blot (c) (n=3 cells), respectively. Statistical analysis of the data was performed using two-sided unpaired t test (a right, b right, c), or 1-way ANOVA (a left, b left) followed by Tukey's multiple comparison tests. Data are presented as means ± SEM from one of three independent experiments. *$P < 0.05$, **$P < 0.01$, ***$P < 0.001$, ****$P < 0.0001$; n.s = not significant.

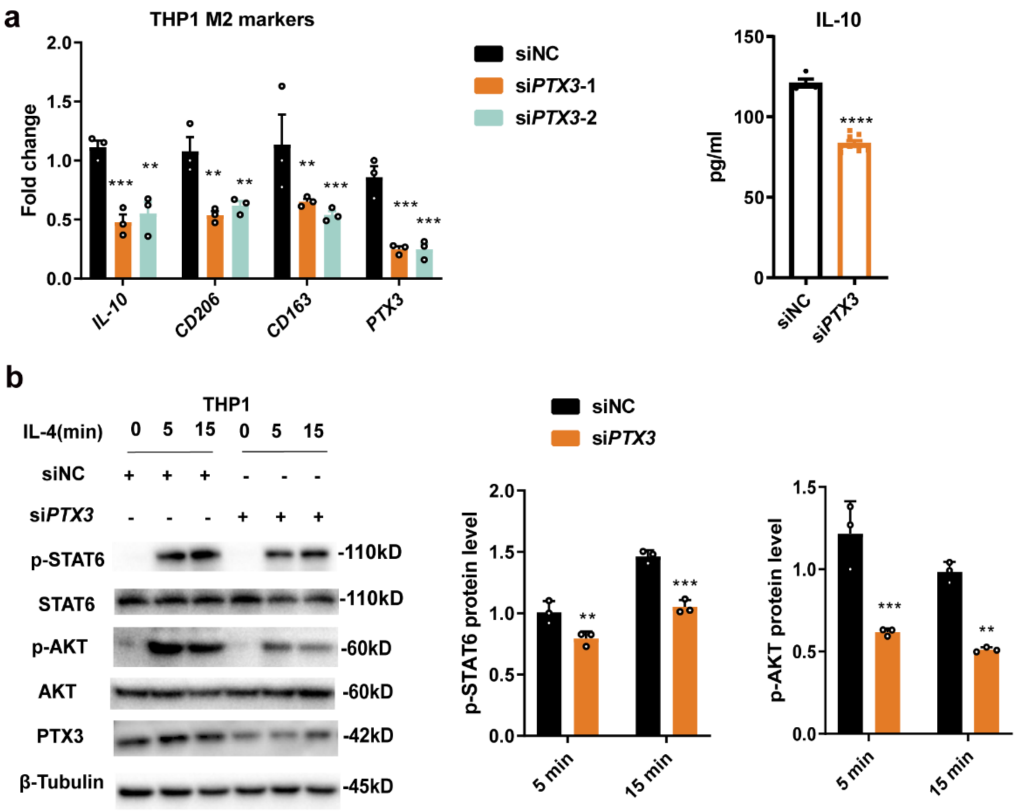

Supplementary Figure 9 PTX3 promotes M2 macrophage activation through the PI3K/AKT and JAK/STAT6 signaling pathways. (a) Knockdown of *PTX3* in human THP1-derived macrophages using two distinct siRNAs (200 nM), following IL-4 stimulation. M2-associated markers were quantified by RT-qPCR (left) (n=3 cells). NC, negative control. ELISA showing the levels of IL-10 secretion (right) (n=4 cells for control group and n=8 cells for knockdown groups). (b) Western blot analysis of AKT and STAT6 phosphorylation in *PTX3*-knockdown THP1-derived macrophages (n=3 cells). Statistical analysis of the data was performed using two-sided unpaired t test (a right), or 2-way ANOVA (a left, b) followed by either Tukey's or Sidak's multiple comparison tests. Data are presented as means ± SEM from one of three independent experiments. $^{**}P < 0.01$, $^{***}P < 0.001$, $^{****}P < 0.0001$.

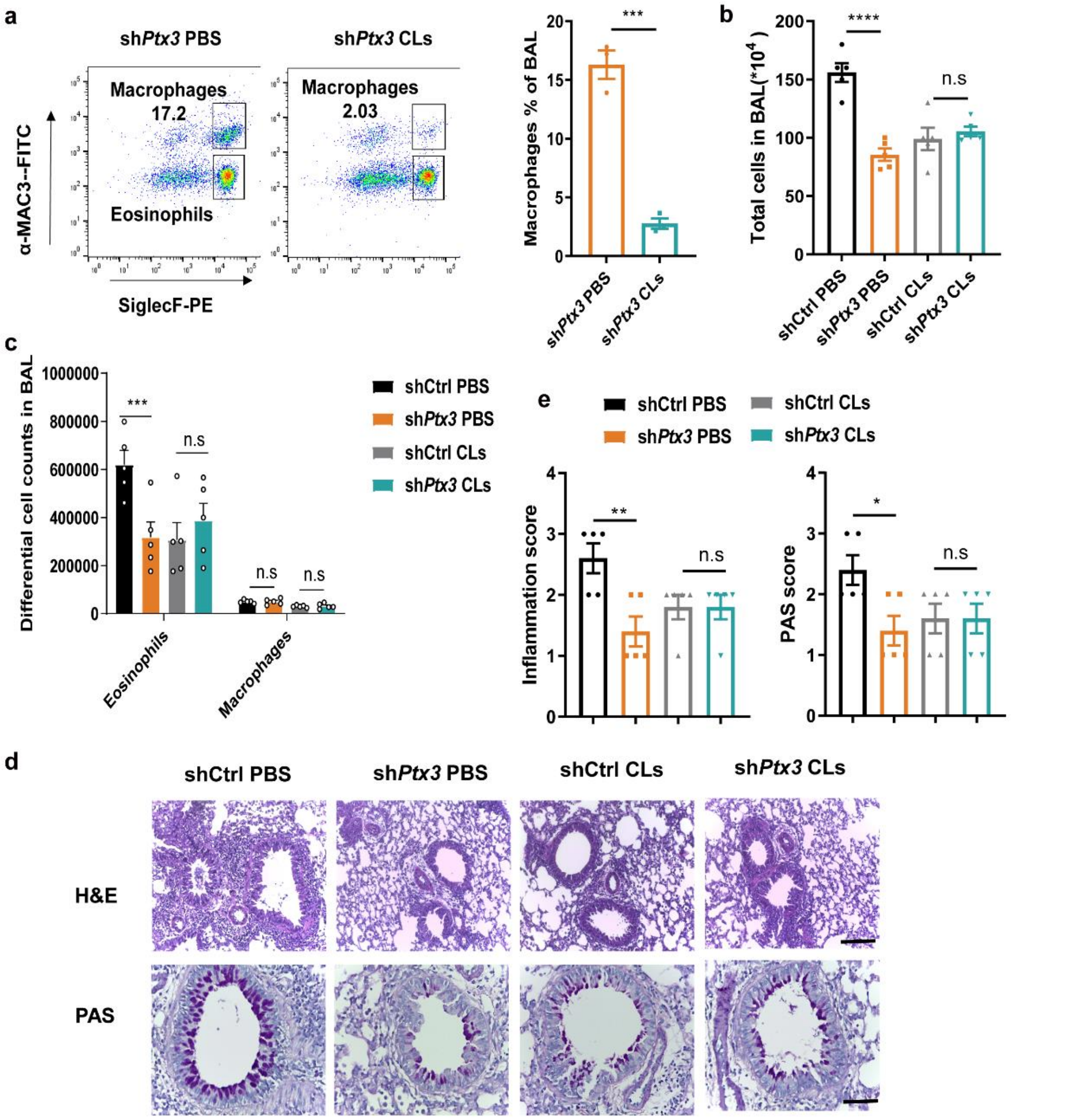

Supplementary Figure 10 The effect of PTX3 on airway inflammation is dependent on macrophages. Every 72 h during CRE treatment, CLs-liposome (200 μl) was i.p. administered in the clodronate group. The sh*Ptx3* lentivirus or shCtrl virus was administered by intratracheal instillation on day 14. (a) Flow cytometry analysis of the efficiency of macrophage depletion in BALF from sh*Ptx3*-treated mice by clodronate treatment (n=3 animals). (b) Total and (c) differential BALF cell numbers from experimental animals were analyzed by flow cytometry (n=5 animals). (d) Histopathological changes in the lung tissues were examined by H&E- and PAS-staining. Scale bars: 200 μm and 100 μm, respectively. (e) Calculated inflammation and PAS scores (n=5 animals). Statistical analysis of the data was performed using two-

sided unpaired t test (a), 1-way ANOVA (b, e), and 2-way ANOVA (c) followed by Tukey's multiple comparison tests. Data are presented as means ± SEM from one of three independent experiments. *$P < 0.05$, **$P < 0.01$, ***$P < 0.001$, ****$P < 0.0001$; n.s = not significant.

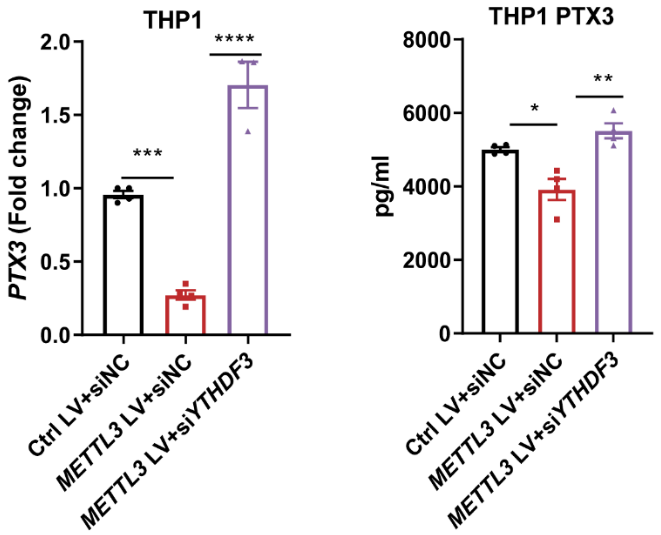

Supplementary Figure 11 METTL3/YTHDF3 axis degrades the *PTX3* expression in human THP1-derived macrophages. *METTL3* or Ctrl LV-treated THP1-derived macrophages were infected with *YTHDF3* siRNAs or control (NC), followed by IL-4 stimulation**.** RT-qPCR and ELISA assays detected the PTX3 mRNA (n=3 for *METTL3* LV+si*YTHDF3* group and n=4 cells for other groups) and protein levels (n=4 cells), respectively. Statistical analysis of the data was performed using 1-way ANOVA followed by Tukey's multiple comparison tests. Data are presented as means ± SEM from one of three independent experiments. *$P < 0.05$, **$P < 0.01$, ***$P < 0.001$, ****$P < 0.0001$.

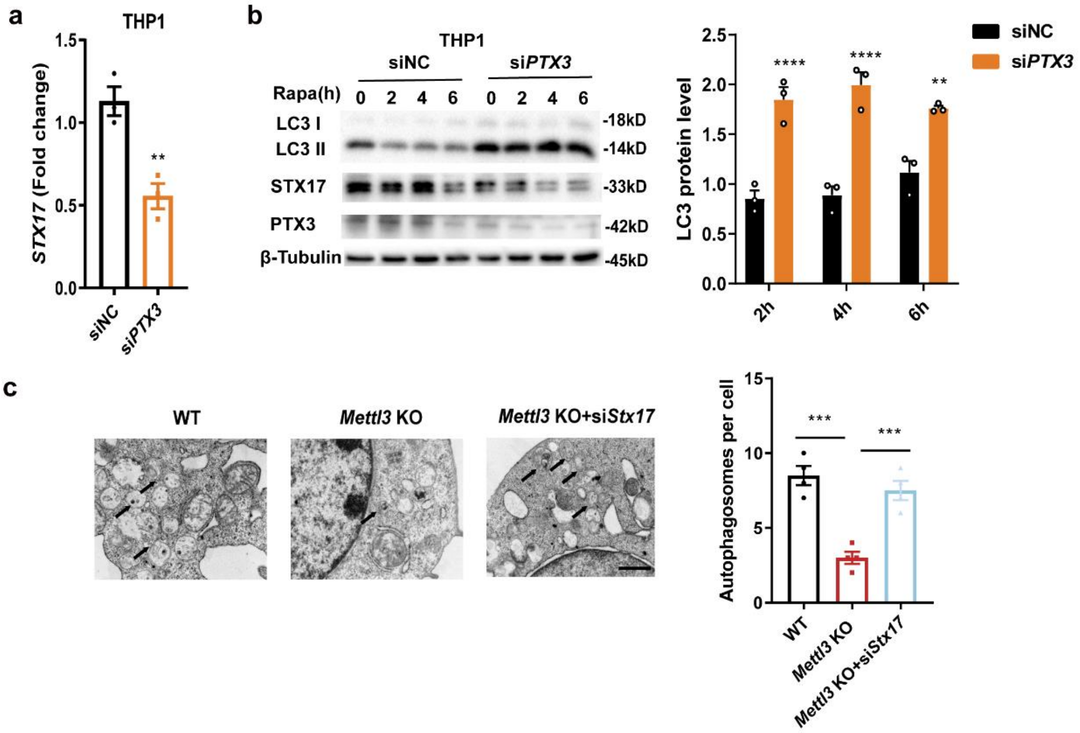

Supplementary Figure 12 METTL3/PTX3/STX17 axis in macrophages controls autophagy maturation. (a) RT-qPCR showing reduced levels of *STX17* in *PTX3*-knockdown THP1-derived macrophages compared to control cells (n=3 cells). (b) Immunoblot analysis of LC3, STX17, and PTX3 levels in control and *PTX3*-knockdown THP1-derived macrophages treated with rapamycin for 0–6 h (n=3 cells). (c) Analysis of the autophagosome number in *Mettl3* KO BMDMs with or without si*Stx17* knockdown (n=4 cells). Scale bars, 1μm. Statistical analysis of the data was performed using two-sided unpaired t test (a), 1-way ANOVA (c), and 2-way ANOVA (b) followed by either Tukey's or Sidak's multiple comparison tests. Data are presented as means ± SEM from one of three independent experiments. $**P < 0.01$, $***P < 0.001$, $****P < 0.0001$.

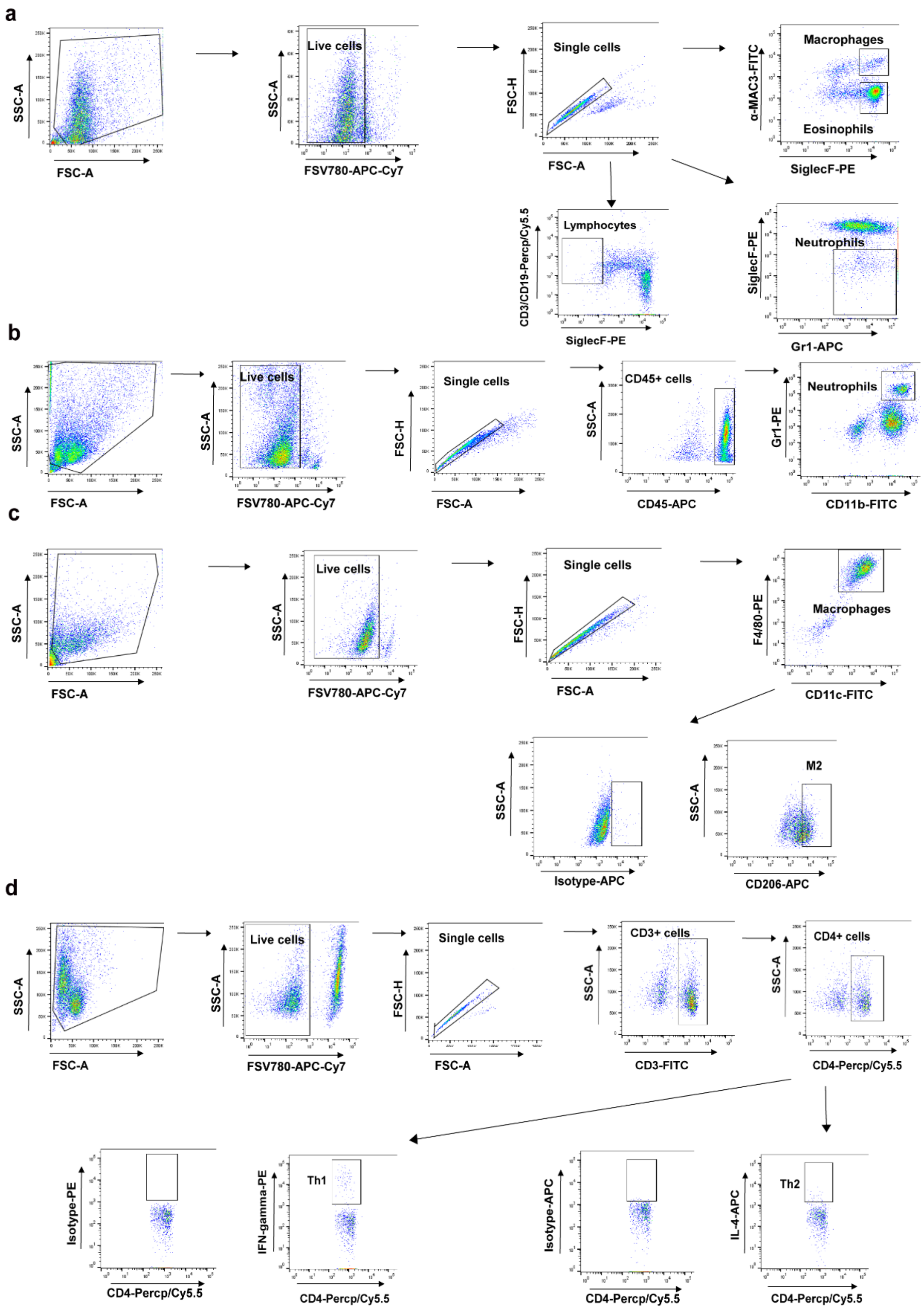

Supplementary Figure 13 Gating strategies used for flow cytometry. (a) Gating strategy to analyze BALF cells, such as eosinophils and macrophages, in experimental models. (b) Gating strategy to analyze the efficiency of neutrophils depletion in BALF

on Supplementary Figure 2. (c) Gating strategy to analyze the M2 macrophage subpopulation in BALF. (d) Gating strategy to analyze the Th2 cells and Th1 cells in MLNs from mice.

Supplementary Table 1. The M2 macrophage activation-associated genes.

M2 macrophage activation-associated genes with known regulatory functions			
STAT6	SOCS1	STAT3	NFIL3
DUSP6	IRF4	PPARG	SOCS2
SOCS3	SBNO2	GATA3	SMAD2
ID3	RGS1	CCL4	CCL13
CCL17	CCL18	CD206	CD163
MMP1	MMP12	IL17RB	TGM2
ALOX15	PTX3	RIPK3	KLF4

Supplementary Table 2. Demographic and respiratory health characteristics of children with allergic asthma and healthy controls.

Characteristic	Normal (N=50)	Asthma(N=55)
**Male/Female, no.**	30/20	34/21
**Age, years**	7.20± 2.67	8.07±2.64
**Asthma duration, years**	N/A	1.40± 1.09
**BMI**	17.68± 3.44	17.52± 2.92
**Blood eos[1] (cells/uL)**	N/A	323.64± 212.28
**Total IgE (ku/L)**	Not done	607.85± 33.41
**C-ACT score [2]**	Not done	22.29± 3.94
**FeNO, ppb**	Not done	23.76± 18.00
**%FEV_1**	Not done	97.23± 18.89

Values are presented as mean ±SEM. [1.] eos, eosinophil. [2.] C-ACT, Childhood Asthma Control Test.

The diagnosis of childhood asthma is established based on combinations of episodic respiratory symptoms (wheezing, cough and dyspnea), reversible airflow limitation, presence of personal or family history of allergic diseases according to the 2016 edition of the Guidelines for the Diagnosis and Prevention of Childhood Bronchial Asthma of China. A lung function test was performed, and percent predicted forced expiratory volume in 1 second (%FEV1), fraction of exhaled nitric oxide (FeNO) levels, blood eosinophil numbers and Childhood Asthma Control Test (C-ACT) scores were recorded. The control group included children of the same age range, who did not suffer from asthma and other allergic diseases. Children with other chronic respiratory conditions, obesity, diabetes, heart disease, immunodeficiency, or any other chronic disease that might impact the main outcomes of this study were excluded. The sex/gender of human participants was determined based on self-reporting. Consent has been obtained for sharing of individual-level data. Patients were not compensated for their participation beyond receipt of therapy and associated care.

Supplementary Table 3. The primer sequences.

Identification of *Mettl3*$^{flox/flox}$ mice

genes	Forward primer(5’-3’)	Reverse primer(5’-3’)
**P1**	GTTTGCACAAGGAGTATTT	TCATCTGGGGAAGGAGGAGTG

Identification of *Lyz2*-Cre mice

genes	Forward primer(5’-3’)	Reverse primer(5’-3’)
**Mutant**	CCCAGAAATGCCAGATTACG	CTTGGGCTGCCAGAATTTCTC
**Wildtype**	CCCAGAAATGCCAGATTACG	TTACAGTCGGCCAGGCTGAC

Mouse RT-qPCR analysis

genes	Forward primer (5’-3’)	Reverse primer (5’-3’)
Actin (beta)	CATTGCTGACAGGATGCAGAAGG	TGCTGGAAGGTGGACAGTGAGG
Il-10	CGGGAAGACAATAACTGCACCC	CGGTTAGCAGTATGTTGTCCAGC
Arg1	CATTGGCTTGCGAGACGTAGAC	GCTGAAGGTCTCTTCCATCACC
Cd206	GTTCACCTGGAGTGATGGTTCTC	AGGACATGCCAGGGTCACCTTT
Tnf	AAACACAAGATGCTGGGACA	TTGATGGTGGTGCATGAGAG
Il-6	TACCACTTCACAAGTCGGAGGC	CTGCAAGTGCATCATCGTTGTTC
Il-1β	GCACTACAGGCTCCGAGATG	CGTTGCTTGGTTCTCCTTGTAC

Ptx3	CGAAATAGACAATGGACTTCATCC	CATCTGCGAGTTCTCCAGCATG
Mettl3	CAGTGCTACAGGATGACGGCTT	CCGTCCTAATGATGCGCTGCAG
Stx17	GCTGCTTCTCAGAGTCTGACTC	TGTCAGTGACCAGATGGCTCAG
Ccl17	CGAGAGTGCTGCCTGGATTACT	GGTCTGCACAGATGAGCTTGCC
Ccl22	GTGGAAGACAGTATCTGCTGCC	AGGCTTGCGGCAGGATTTTGAG
Ifng	CAGCAACAGCAAGGCGAAAAAGG	TTTCCGCTTCCTGAGGCTGGAT
Il-17a	CAGACTACCTCAACCGTTCCAC	TCCAGCTTTCCCTCCGCATTGA
Il-4	ATCATCGGCATTTTGAACGAGGTC	ATCATCGGCATTTTGAACGAGGTC
Il-13	AACGGCAGCATGGTATGGAGTG	TGGGTCCTGTAGATGGCATTGC

Human RT-qPCR analysis

genes	Forward primer (5'-3')	Reverse primer (5'-3')
ACTIN	CACCATTGGCAATGAGCGGTTC	AGGTCTTTGCGGATGTCCACGT
METTL3	CTATCTCCTGGCACTCGCAAGA	GCTTGAACCGTGCAACCACATC
IL-10	TCTCCGAGATGCCTTCAGCAGA	TCAGACAAGGCTTGGCAACCCA
CD163	CCAGAAGGAACTTGTAGCCACAG	CAGGCACCAAGCGTTTTGAGCT

CD206	AGCCAACACCAGCTCCTCAAGA	CAAAACGCTCGCGCATTGTCCA
YTHDF3	GCTACTTTCAAGCATACCACCTC	ACAGGACATCTTCATACGGTTATTG
PTX3	CGAAATAGACAATGGACTCCATCC	CTCATCTGCGAGTTCTCCAGCA
METTL14	CTGAAAGTGCCGACAGCATTGG	CTCTCCTTCATCCAGATACTTACG
FTO	CCAGAACCTGAGGAGAGAATGG	CGATGTCTGTGAGGTCAAACGG
ALKBH5	CCAGCTATGCTTCAGATCGCCT	GGTTCTCTTCCTTGTCCATCTCC
TNF	CTCTTCTGCCTGCTGCACTTTG	ATGGGCTACAGGCTTGTCACTC
IL-6	AGACAGCCACTCACCTCTTCAG	TTCTGCCAGTGCCTCTTTGCTG
IL-1β	CCACAGACCTTCCAGGAGAATG	GTGCAGTTCAGTGATCGTACAGG
STX17	TCGTGGGAAACCTTAGAAGCGG	GCAGCACTGTTGACATGGTCTG

SELECT qPCR analysis

genes	Up primer (5'-3')	Down primer (5'-3')
PTX3 **AGA^{1333}CT**	tagccagtaccgtagtgcgtgGAGTTTCTTTCTTTGGCTTCAAGTGGAG	5phos/TTCACAACATTTATGAAACATACTGAGCTCcagaggctgagtcgctgcat

Gene-specific m^{6}A qPCR analysis

genes	Forward primer (5'-3')	Reverse primer (5'-3')

PTX3	CAGCCACATGGAGGAGCTCAGTATGTTTCA	TCAACTGTTTATTCAGTATTTGGCCAGTAC

Human *PTX3* 3’UTR dual-luciferase reporter analysis

**genes**	**Forward primer(5’-3’)**	**Reverse primer(5’-3’)**
***PTX3* WT 3’UTR**	CATCGAGAAACTCCACTTGAAGCCAAAGAAAGAAAC	GGCTACTTCAACTGTTTATTCAGTATTTGGCCAGTA
***PTX3* Mutant 3’UTR**	TAATAGGAACACTTGAGTCTAATGAAAGAGAGAGT	ACTCTCTCTTTCATTAGACTCAAGTGTTCCTATTA

Human *METTL3* lentivirus construct

**genes**	**Forward primer (5’-3’)**	**Reverse primer (5’-3’)**
***METTL3*-1**	AGGGTTCCAAGCTTAAGCGGCCGCGCCACCATGTCGGACACGTGGAGCTCTATCCAG	
***METTL3*-2**	ATCAGTAGAGAGTGTCGGATCCCTATAAATTCTTAGGTTTAGAGATGATGCCGTCC	

Supplementary Table 4. The sequences of siRNA and shRNA lentivirus.

GENES	Sense (5'-3')	Antisense (5'-3')
**Human NC siRNA**	UUCUCCGAACGUGUCACGUTT	ACGUGACACGUUCGGAGAATT
**Human *METTL3* siRNA1**	GCAGAACAGGACUCGACUATT	UAGUCGAGUCCUGUUCUGCTT
**Human *METTL3* siRNA2**	GCUCAACAUACCCGUACUATT	UAGUACGGGUAUGUUGAGCTT
**Human *PTX3* siRNA1**	GCACAAAGAGGAAUCCAUATT	UAUGGAUUCCUCUUUGUGCTT
**Human *PTX3* siRNA2**	GGGAUAGUGUUCUUAGCAATT	UUGCUAAGAACACUAUCCCTT
**Human *YTHDF3* siRNA1**	AGCAGAGGAAACAGGCGAATT	UUCGCCUGUUUCCUCUGCUTT
**Human *YTHDF3* siRNA2**	GCUGGAUUUGGCAAUGAUATT	UAUCAUUGCCAAAUCCAGCTT
**Mouse shCtrl**	TTCTCCGAACGTGTCACGT	
**Mouse sh*Ptx3*-1**	GAGCTCATGTATGTGAATTTG	
**Mouse sh*Ptx3*-2**	GGGACAAGCTGTTCATCATGC	
**Mouse**	GAGCCAGCGAUACAGAAA	

**sh*Stx17*-1**	UTT
**Mouse**	GCUCCAAUAUCCGAGAAA
**sh*Stx17*-2**	UTT
