# Supplementary material for: RNA m6A methylation modulates airway inflammation in allergic asthma via PTX3-dependent macrophage homeostasis
Source: Nat Commun. 2023 Nov 13;14:7328. doi: 10.1038/s41467-023-43219-w (PMC10643624; doi:10.1038/s41467-023-43219-w)
Supplement: Supplementary file 3 — Reporting Summary [file 41467_2023_43219_MOESM3_ESM.pdf]

nature portfolio

Corresponding author(s): Yufeng Zhou, NCOMMS-22-37321B

Last updated by author(s): Oct 13, 2023

Reporting Summary

Nature Portfolio wishes to improve the reproducibility of the work that we publish. This form provides structure for consistency and transparency in reporting. For further information on Nature Portfolio policies, see our Editorial Policies and the Editorial Policy Checklist.

Statistics

For all statistical analyses, confirm that the following items are present in the figure legend, table legend, main text, or Methods section.

n/a	Confirmed	
☐	☒	The exact sample size (n) for each experimental group/condition, given as a discrete number and unit of measurement
☐	☒	A statement on whether measurements were taken from distinct samples or whether the same sample was measured repeatedly
☐	☒	The statistical test(s) used AND whether they are one- or two-sided<br>*Only common tests should be described solely by name; describe more complex techniques in the Methods section.*
☒	☐	A description of all covariates tested
☐	☒	A description of any assumptions or corrections, such as tests of normality and adjustment for multiple comparisons
☐	☒	A full description of the statistical parameters including central tendency (e.g. means) or other basic estimates (e.g. regression coefficient) AND variation (e.g. standard deviation) or associated estimates of uncertainty (e.g. confidence intervals)
☐	☒	For null hypothesis testing, the test statistic (e.g. F, t, r) with confidence intervals, effect sizes, degrees of freedom and P value noted<br>*Give P values as exact values whenever suitable.*
☒	☐	For Bayesian analysis, information on the choice of priors and Markov chain Monte Carlo settings
☒	☐	For hierarchical and complex designs, identification of the appropriate level for tests and full reporting of outcomes
☒	☐	Estimates of effect sizes (e.g. Cohen's d, Pearson's r), indicating how they were calculated

Our web collection on statistics for biologists contains articles on many of the points above.

Software and code

Policy information about availability of computer code

Data collection	For m6A -seq assay, both input and m6A IP samples were deeply sequenced on the Illumina Hiseq 4000 at OE Biotech. Co. Ltd (Shanghai, China). For RNA-seq assay, samples were sequenced on Illumina NovaSeq 6000 instrument at Sinotech Genomics. Co. Ltd (Shanghai, China). Flow cytometry data was acquired with BD FACSCanto II. RT-qPCR data was acquired with Roche LC 480. Western blotting data was collected using Bio-Rad ChemiDoc XRS+. Immunofluorescence images were acquired with Leica TSC SP8. Measurements of airway hyperresponsiveness were made on a Buxco FinePointe Resistance and Compliance System. For ELISA and luminescence measurements, a Thermo Scientific Varioskan™ LUX was used. TAM data was collected using Hitachi HT-7800 transmission electron microscope.
Data analysis	For m6A -seq assay, the differentially expressed transcripts of m6A methylome between METTL3-knockdown and Ctrl macrophages were detected by MeTDiffpeak (version 1.1.0) software. For RNA-seq assay, differential genes expression were analyzed by the standard Illumina sequence analysis pipeline. Flow cytometry data was analyzed with FlowJoV10 software. Immunofluorescence and Western blotting images were analyzed with Image J 1.8 software. Airway resistance was calculated using Buxco FinePointe software. The statistical analyses were performed using GraphPad Prism 9.0 software.

For manuscripts utilizing custom algorithms or software that are central to the research but not yet described in published literature, software must be made available to editors and reviewers. We strongly encourage code deposition in a community repository (e.g. GitHub). See the Nature Portfolio guidelines for submitting code & software for further information.

Data

Policy information about availability of data

All manuscripts must include a data availability statement. This statement should provide the following information, where applicable:

- Accession codes, unique identifiers, or web links for publicly available datasets
- A description of any restrictions on data availability
- For clinical datasets or third party data, please ensure that the statement adheres to our policy

The raw data from the RNA-Seq and m6A-Seq have been deposited in the GEO database under accession numbers GSE192533, GSE192726, and GSE193340 (public), respectively. The accessible links of GSE27876 used in the study are provided in the Supplementary Information. Uncropped and unprocessed scans of blots have been provided as in the Source data file. All data needed to evaluate the conclusions in the paper are present in the paper and the Supplementary Information. Source data are provided with this paper.

Research involving human participants, their data, or biological material

Policy information about studies with human participants or human data. See also policy information about sex, gender (identity/presentation), and sexual orientation and race, ethnicity and racism.

Reporting on sex and gender	The sex/gender of human participants was determined based on self-reporting. The human participants providing samples for our study were broadly age- and sex-matched and are detailed in Supplementary Information Table 2. Consent has been obtained for sharing of individual-level data. Compared with girls, boys have a higher incidence of asthma during childhood, while women as adults have an increased prevalence and severity of asthma (PMID: 34789462；PMID: 30619350). A nationwide survey of childhood asthma in urban areas of China also found that the prevalence of asthma in boys (3.51%) were higher than girls (2.29%), however the survey of adult asthma in China didn't find the prevalence difference between men (4.6%) and women (3.7%) (PMID: 24406223; PMID: 31230828). Due to the small sample size, our study was not sufficiently powered to detect differences according to sex/gender.
Reporting on race, ethnicity, or other socially relevant groupings	Only Chinese patients and health controls are included.
Population characteristics	Characteristic Normal (N=50) Asthma(N=55)<br>Male/Female, no. 30/20 34/21<br>Age, years 7.20± 2.67 8.07±2.64<br>Asthma duration, years N/A 1.40± 1.09<br>BMI 17.68± 3.44 17.52± 2.92<br>Blood eos1 (cells/uL) N/A 323.64± 212.28<br>Total IgE (ku/L) Not done 607.85± 33.41<br>C-ACT score 2 Not done 22.29± 3.94<br>FeNO, ppb Not done 23.76± 18.00<br>%FEV1 Not done 97.23± 18.89
Recruitment	55 children with allergic asthma and 50 age and sex-matched healthy controls were recruited from the Children's Hospital of Fudan University. The diagnosis of childhood asthma is established based on combinations of episodic respiratory symptoms (wheezing, cough, and dyspnea), reversible airflow limitation, presence of personal or family history of allergic diseases according to the 2016 edition of the Guidelines for the Diagnosis and Prevention of Childhood Bronchial Asthma of China. A lung function test was performed, and percent predicted forced expiratory volume in 1 second (%FEV1), fraction of exhaled nitric oxide (FeNO) levels, blood eosinophil numbers and Childhood Asthma Control Test (C-ACT) scores were recorded. The control group included children of the same age range, who did not suffer from asthma and other allergic diseases. Children with other chronic respiratory conditions, obesity, diabetes, heart disease, immunodeficiency, or any other chronic disease that might impact the main outcomes of this study were excluded.<br>Informed consent was obtained from the parents of all participants.
Ethics oversight	The study was approved by the Research Ethics Board of the Children's Hospital of Fudan University (No. 2020-81).

Note that full information on the approval of the study protocol must also be provided in the manuscript.

Field-specific reporting

Please select the one below that is the best fit for your research. If you are not sure, read the appropriate sections before making your selection.

[x] Life sciences [ ] Behavioural & social sciences [ ] Ecological, evolutionary & environmental sciences

For a reference copy of the document with all sections, see nature.com/documents/nr-reporting-summary-flat.pdf

Life sciences study design

All studies must disclose on these points even when the disclosure is negative.

Sample size	Statistical methods were not used to predetermine sample size. Sample size was determined based on common standards for experimental cell biology and animal studies, attempting to have a minimum of n=3 biological replicates with sufficient reproducibility. In the in vivo assays, the number of animals used for each experiment was estimated based on previous and pilot studies. 4-6 mice for each experimental group in this study were analyzed to ensure the differences. In the in vitro assays, variability used in this study tends to be relatively low, so we used >= 3 independent biological replicates.
Data exclusions	No data were excluded from the analysis.
Replication	Data supporting the conclusions of the study are presented as means ± SEM from one of three independent experiments, with at least three independent biological replicates per experimental group. All attempts to reproduce the results were successful. This was indicated in the Figure legends.
Randomization	For in vitro assays, samples were randomly allocated into the different experimental groups. Age-matched mice were randomly allocated into the experimental groups on day 0 before treatment application.
Blinding	For IHC and IF assays, the operator analyzing the image was blinded. Animal assays were not blinded, since they were in vivo studies where the treatment groups needed to be clear when performing the experiments. For the in vitro assays, no blinding was used as we treated and analyzed the samples in a similar procedure.

Reporting for specific materials, systems and methods

We require information from authors about some types of materials, experimental systems and methods used in many studies. Here, indicate whether each material, system or method listed is relevant to your study. If you are not sure if a list item applies to your research, read the appropriate section before selecting a response.

Materials & experimental systems

n/a	Involved in the study	
☐	☒	Antibodies
☐	☒	Eukaryotic cell lines
☒	☐	Palaeontology and archaeology
☐	☒	Animals and other organisms
☒	☐	Clinical data
☒	☐	Dual use research of concern
☒	☐	Plants

Methods

n/a	Involved in the study	
☒	☐	ChIP-seq
☐	☒	Flow cytometry
☒	☐	MRI-based neuroimaging

Antibodies

Antibodies used

Antibodies used in western blotting:
Phospho-Stat6 (Tyr641) Antibody #9361, 1:1000, Cell Signaling Technology
Stat6 Antibody #9362, 1:1000, Cell Signaling Technology
Phospho-Akt (Ser473) (D9E) XP® Rabbit mAb #4060, 1:1000, Cell Signaling Technology
Akt (pan) (40D4) Mouse mAb #2920, 1:1000, Cell Signaling Technology
METTL3 (D2I6O) Rabbit mAb #96391, 1:1000, Cell Signaling Technology
Pentraxin 3 Polyclonal antibody Cat no: 13797-1-AP, 1:1000, Proteintech
YTHDF3-specific Rabbit Polyclonal antibody Cat no: 25537-1-AP, 1:1000, Proteintech
LC3 Polyclonal antibody Cat no: 14600-1-AP, 1:1000, Proteintech
Syntaxin 17 Rabbit Polyclonal Antibody Cat no: 17815-1-AP, 1:1000, Proteintech
Anti-Mannose Receptor/CD206 antibody (ab64693), 1:1000, Abcam
Anti-beta Tubulin antibody - Loading Control (ab6046), 1:5000, Abcam
Anti-rabbit IgG, HRP-linked Antibody #7074, 1:5000, Cell Signaling Technology
Anti-mouse IgG, HRP-linked Antibody #7076, 1:5000, Cell Signaling Technology

Antibodies used in FACS:
Fixable Viability Stain 780, Cat no: 565388, 1:1000, BD
CD16/CD32 Monoclonal Antibody (93), Cat no: 14-0161-82, 1:100, eBioscience
SiglecF-PE, Cat no: 155505, 1:100, BioLegend
MAC-3-FITC, Cat no: 108504, 1:200, BioLegend
Gr-1-APC, Cat no: 108412, 1:200, BioLegend
CD19-PerCP/Cy5.5, Cat no: 152405, 1:250, BioLegend
CD3-PerCP/Cy5.5, Cat no: 100327, 1:100, BioLegend

CD45-APC, Cat no: 103111, 1:100, BioLegend
CD11b-FITC, Cat no: 101205, 1:200, BioLegend
Gr-1-PE, Cat no: 127607, 1:200, BioLegend
CD11c-FITC, Cat no: 117305, 1:100, BioLegend
F4//80-PE, Cat no: 123109, 1:200, BioLegend
CD206-APC, Cat no: 141707, 1:40, BioLegend
CD3-FITC, Cat no: 100203, 1:50, BioLegend
CD4- PerCP/Cy5.5, Cat no: 100433, 1:80, BioLegend
IL-4-APC, Cat no: 504105, 1:20, BioLegend
IFN-gamma-PE, Cat no: 163503, 1:40, BioLegend

Antibodies used in Immunohistochemistry:
Ly-6G (E6Z1T) Rabbit mAb #87048, 1:50, Cell Signaling Technology

Antibodies used in Immunofluorescent staining:
F4/80 (D2S9R) XP® Rabbit mAb #70076, 1:250, Cell Signaling Technology
Anti-Mannose Receptor/CD206 antibody (ab64693), 1:200, Abcam
Pentraxin 3 Polyclonal antibody Cat no: 13797-1-AP, 1:100, Proteintech

Antibodies used in MeRIP-qPCR and RIP:
Anti-N6-methyladenosine Antibody (m6A), clone 17-3-4-1, Cat no: MABE1006, 2μg /ml, Millipore
YTHDF3 Antibody (F-2), sc-377119, 2μg /ml, Santa Cruz

Validation

All antibodies were manufacturer validated and commercially available. Validation statements are available on the manufacturer's website. We also validated these antibodies in the preliminary experiment to confirm the concentration and specific binding, as well as the suitable blocking reagent.

Antibodies used in western blotting:
Phospho-Stat6 (Tyr641) Antibody #9361, 1:1000, Cell Signaling Technology, 5% BSA/TBS, React: mouse, human
Stat6 Antibody #9362, 1:1000, Cell Signaling Technology, 5% BSA/TBS, React: mouse, human
Phospho-Akt (Ser473) (D9E) XP® Rabbit mAb #4060, 1:1000, Cell Signaling Technology, 5% BSA/TBS, React: mouse, human
Akt (pan) (40D4) Mouse mAb #2920, 1:1000, Cell Signaling Technology, 5% BSA/TBS, React: mouse, human
METTL3 (D2I6O) Rabbit mAb #96391, 1:1000, Cell Signaling Technology, 5% BSA/TBS, React: mouse, human
Pentraxin 3 Polyclonal antibody Cat no: 13797-1-AP, 1:1000, Proteintech, 5% BSA/TBS, React: mouse, human
YTHDF3-specific Rabbit Polyclonal antibody Cat no: 25537-1-AP, 1:1000, Proteintech, 5% BSA/TBS, React: mouse, human
LC3 Polyclonal antibody Cat no: 14600-1-AP, 1:1000, Proteintech, 5% BSA/TBS, React: mouse, human
Syntaxin 17 Rabbit Polyclonal Antibody Cat no: 17815-1-AP, 1:1000, Proteintech, 5% BSA/TBS, React: mouse, human
Anti-Mannose Receptor/CD206 antibody (ab64693), 1:1000, Abcam, 5% BSA/TBS, React: mouse, human
Anti-beta Tubulin antibody - Loading Control (ab6046), 1:5000, Abcam, 5% BSA/TBS, React: mouse, human
Anti-rabbit IgG, HRP-linked Antibody #7074, 1:5000, Cell Signaling Technology
Anti-mouse IgG, HRP-linked Antibody #7076, 1:5000, Cell Signaling Technology, 5% BSA/TBS

Antibodies used in FACS:
Fixable Viability Stain 780, Cat no: 565388, 1:1000, BD, DPBS, React: mouse
CD16/CD32 Monoclonal Antibody (93), Cat no: 14-0161-82, 1:100, eBioscience, 2%FBS/PBS, React: mouse
SiglecF-PE, Cat no: 155505, 1:100, BioLegend, 2%FBS/PBS, React: mouse
MAC-3-FITC, Cat no: 108504, 1:200, BioLegend, 2%FBS/PBS, React: mouse
Gr-1-APC, Cat no: 108412, 1:200, BioLegend, 2%FBS/PBS, React: mouse
CD19-PerCP/Cy5.5, Cat no: 152405, 1:250, BioLegend, 2%FBS/PBS, React: mouse
CD3-PerCP/Cy5.5, Cat no: 100327, 1:100, BioLegend, 2%FBS/PBS, React: mouse
CD45-APC, Cat no: 103111, 1:100, BioLegend, 2%FBS/PBS, React: mouse
CD11b-FITC, Cat no: 101205, 1:200, BioLegend, 2%FBS/PBS, React: mouse
Gr-1-PE, Cat no: 127607, 1:200, BioLegend, 2%FBS/PBS, React: mouse
CD11c-FITC, Cat no: 117305, 1:100, BioLegend, 2%FBS/PBS, React: mouse
F4//80-PE, Cat no: 123109, 1:200, BioLegend, 2%FBS/PBS, React: mouse
CD206-APC, Cat no: 141707, 1:40, BioLegend, 2%FBS/PBS, React: mouse
CD3-FITC, Cat no: 100203, 1:50, BioLegend, 2%FBS/PBS, React: mouse
CD4- PerCP/Cy5.5, Cat no: 100433, 1:80, BioLegend, 2%FBS/PBS, React: mouse
IL-4-APC, Cat no: 504105, 1:20, BioLegend, 2%FBS/PBS, React: mouse
IFN-gamma-PE, Cat no: 163503, 1:40, BioLegend, 2%FBS/PBS, React: mouse

Antibodies used in Immunohistochemistry:
Ly-6G (E6Z1T) Rabbit mAb #87048, 1:50, Cell Signaling Technology, 5% BSA/PBS, React: mouse

Antibodies used in Immunofluorescent staining:
F4/80 (D2S9R) XP® Rabbit mAb #70076, 1:250, Cell Signaling Technology, 5% BSA/PBS, React: mouse
Anti-Mannose Receptor/CD206 antibody (ab64693), 1:200, Abcam, 5% BSA/PBS, React: mouse

Pentraxin 3 Polyclonal antibody Cat no: 13797-1-AP, 1:100, Proteintech, 5% BSA/PBS, React: mouse

Antibodies used in MeRIP-qPCR and RIP:
Anti-N6-methyladenosine Antibody (m6A), clone 17-3-4-1, Cat no: MABE1006, 2µg /ml, Millipore, React: mouse, human
YTHDF3 Antibody (F-2), sc-377119, 2µg /ml, Santa Cruz, React: mouse, human

Eukaryotic cell lines

Policy information about cell lines and Sex and Gender in Research

Cell line source(s)	THP1 cells were purchased from the American Type Culture Collection (ATCC).
Authentication	Cell lines were authenticated using the profiles of short tandem repeats reported within the last 3 years.
Mycoplasma contamination	All cell lines were tested to be mycoplasma negative.
Commonly misidentified lines (See ICLAC register)	No cell line listed by ICLAC was used.

Animals and other research organisms

Policy information about studies involving animals; ARRIVE guidelines recommended for reporting animal research, and Sex and Gender in Research

Laboratory animals	All mice were housed, bred, and maintained under specific pathogen-free conditions, fed standard laboratory chow, and kept on a 12h light/dark cycle, and temperature and humidity were kept at 22±1°C, 55%±5%. All mice were on the C57BL/6 genetic background, maintained in individual cages, and used between 6 and 8 weeks of age. Co-housed Cre-negative littermate mice were used as control animals in all experiments.
Wild animals	The study did not involve wild animals.
Reporting on sex	Experiments involving asthma models were conducted in female mice since female mice are more susceptible to the development of allergic airway inflammation than male mice observed in previous studies (PMID: 18066124; PMID: 16297148; PMID: 20413985).
Field-collected samples	The study did not involve field-collected samples.
Ethics oversight	Experiments complied with the relevant laws and institutional guidelines, as overseen by the Animal Studies Committee of the Children's Hospital of Fudan University.

Note that full information on the approval of the study protocol must also be provided in the manuscript.

Plants

Seed stocks	N/A
Novel plant genotypes	N/A
Authentication	N/A

Flow Cytometry

Plots

Confirm that:

- [x] The axis labels state the marker and fluorochrome used (e.g. CD4-FITC).
- [x] The axis scales are clearly visible. Include numbers along axes only for bottom left plot of group (a 'group' is an analysis of identical markers).
- [x] All plots are contour plots with outliers or pseudocolor plots.
- [x] A numerical value for number of cells or percentage (with statistics) is provided.

Methodology

Sample preparation	Mice were sacrificed and Bronchoalveolar lavage fluid (BALF) was harvested by two consecutive flushes of the lung with 1 ml ice-cold PBS. Lavage fluids were centrifuged at 400g, 4°C for 5 min and washed with PBS containing 2% FBS. For M2 macrophage subpopulation analysis, BALF cells were further purified by macrophage adherence and stimulated by IL-4 for 12 h. For Th1/Th2-associated intracellular cytokine staining, cells isolated from mediastinal lymph nodes (MLNs) were stimulated with phorbol myristate acetate (PMA) and ionomycin for 8 h in the presence of brefeldin A and monensin. Cells were stained with Fixable Viability Stain 780 and incubated with an anti-CD16/32 monoclonal antibody. Then, cells were stained with surface markers antibodies, fixed and permeabilized, followed by incubated with isotype control and various cytokine antibodies, respectively.
Instrument	Cells were analyzed on a BD FACSCanto II.
Software	Analysis of flow cytometry data was performed using FlowJoV10 software
Cell population abundance	The purified alveolar macrophages detected with purity >90% were used.
Gating strategy	The live and single cell populations were determined by SSC-A/FSV 780-APC-Cy7 and FSC-H/FSC-A gate, respectively. The boundaries were determined by the clear cell subpopulations and isotype controls.

☒ Tick this box to confirm that a figure exemplifying the gating strategy is provided in the Supplementary Information.
